# Bisdemethoxycurcumin (BDC)-Loaded H-Ferritin-Nanocages Mediate the Regulation of Inflammation in Alzheimer’s Disease Patients

**DOI:** 10.3390/ijms23169237

**Published:** 2022-08-17

**Authors:** Stella Gagliardi, Marta Truffi, Veronica Tinelli, Maria Garofalo, Cecilia Pandini, Matteo Cotta Ramusino, Giulia Perini, Alfredo Costa, Sara Negri, Serena Mazzucchelli, Arianna Bonizzi, Leopoldo Sitia, Maria Busacca, Marta Sevieri, Michela Mocchi, Alessandra Ricciardi, Davide Prosperi, Fabio Corsi, Cristina Cereda, Carlo Morasso

**Affiliations:** 1 IRCCS Mondino Foundation, 27100 Pavia, Italy; 2 Istituti Clinici Scientifici Maugeri IRCCS Spa SB, 27100 Pavia, Italy; 3 Department of Biotechnology and Bioscience, University of Milano-Bicocca, 20126 Milano, Italy; 4 Department of Brain and Behavioral Sciences, University of Pavia, 27100 Pavia, Italy; 5 Department of Biomedical and Clinical Sciences, Università degli Studi di Milano, Via G. B. Grassi 74, 20157 Milano, Italy

**Keywords:** bisdemethoxycurcumin, H-Ferritin, Alzheimer’s disease, gene expression, inflammation, curcumin, drug delivery, nanoparticles

## Abstract

Background: Bisdemethoxycurcumin (BDC) might be an inflammation inhibitor in Alzheimer’s Disease (AD). However, BDC is almost insoluble in water, poorly absorbed by the organism, and degrades rapidly. We thus developed a new nanoformulation of BDC based on H-Ferritin nanocages (BDC-HFn). Methods: We tested the BDC-HFn solubility, stability, and ability to cross a blood–brain barrier (BBB) model. We tested the effect of BDC-HFn on AD and control (CTR) PBMCs to evaluate the transcriptomic profile by RNA-seq. Results: We developed a nanoformulation with a diameter of 12 nm to improve the solubility and stability. The comparison of the transcriptomics analyses between AD patients before and after BDC-HFn treatment showed a major number of DEG (2517). The pathway analysis showed that chemokines and macrophages activation differed between AD patients and controls after BDC-HFn treatment. BDC-HFn binds endothelial cells from the cerebral cortex and crosses through a BBB in vitro model. Conclusions: Our data showed how BDC-Hfn could improve the stability of BDC. Significant differences in genes associated with inflammation between the same patients before and after BDC-Hfn treatment have been found. Inflammatory genes that are upregulated between AD and CTR after BDC-HFn treatment are converted and downregulated, suggesting a possible therapeutic approach.

## 1. Introduction

Alzheimer’s disease (AD) is a chronic neurodegenerative disorder that represents the most common type of dementia and is characterized by the presence of deposits of the protein fragment beta-amyloid (Aβ) and twisted fibers of the protein tau (tangles) in the brain [1]. Different pathways have been proven to be causative of AD. Among the other factors, genetics, inflammation, immune system, oxidative stress, and autophagy are known to be involved [2]. Inflammation in AD patients has a fundamental role. In fact, elevated levels of inflammatory markers in patients with AD have been described, such as risk genes associated with AD related to innate immune functions and inflammation [3].

Indeed, different papers have described the alteration of proinflammatory cytokines such as interleukins (IL-1β, IL-6, and IL-18); tumor necrosis factor (TNF); and chemokines such as C-C motif chemokine ligands (CCL1, CCL5, and CXCL1) [3,4]. Microglial activation releases cytokines and neurotoxic agents such as IL-1β, TNF, IL-6, nitric oxide, and reactive oxygen species (ROS) that can cause neuronal cell death, while Aβ deposition is one of the main biological features of AD [5,6]. Even RNA regulation has been recently associated with inflammation in AD; in particular, noncoding RNA may act as an inhibitor of the NF-κB signaling pathway and activator of inflammatory mediators [7].

As reported in the literature, AD also affects the peripheral tissues, such as blood cells, including peripheral blood mononuclear cells (PBMCs). In fact, it has been shown that PBMCs from AD patients share alterations that have been found in other tissues, including neurons [8,9,10]. PBMCs have been often used as a model to investigate neurodegenerative diseases, because, even if they are peripheral cells, they offer the possibility to study and regulate pathways associated with neurodegenerative disorders as mitochondrial disfunctions, oxidative stress [11,12,13], and epigenetic [14].

Based on these data, different investigations have now been focused on the study of PBMCs, which more specifically reflect inflammatory and apoptotic mechanisms in AD, as we describe in the current paper.

Additionally, different studies have been published about PBMCs from patients treated with different molecules. In fact, we have already published one work regarding monocytes cells treated with more than 45 curcuminoids, and next, we have tested the better curcuminoids between the 45 analyzed in PBMCs from AD patients and matched controls [15].

Moreover, before us, Fiala and collaborators already used PBMCs as a model for AD treatment [16]. So far, medications available to treat AD address symptomatic issues. Agents targeting inflammation, neurotransmission, and Aβ degradation usually show modest clinical benefits [17]. In this scenario, natural supplements targeting the accumulation of amyloids are a promising approach to preventing dementia, linked with the increasing awareness of an important healthy nutrition [18,19]. Among the natural products, several groups reported Curcuma longa (CL) as an up-and-coming agent with proven pharmacological effects on AD, both in vitro and in animal models [20,21,22].

Curcumin is a polyphenolic compound derived from the dried rhizome of CL. In general, curcuminoids have been shown to have different pharmacological properties for neuroprotection and as anti-inflammatory agents. It is indeed well-known that curcumin act as a reactive oxygen species scavenger and limits lipid peroxidation [23,24]. A few reports also suggest that curcumin can protect the integrity of the BBB [25,26]. Additionally, curcuminoids improve the degradation of accumulated Aβ [19,27,28].

Different curcumin analogs have been synthesized, such as bisdemethoxycurcumin (BDC). Previous works demonstrated that BDC is more potent than curcumin as an anti-inflammatory, antiproliferative agent and more powerful in inducing ROS [21,29].

Regarding inflammation, BDC pretreatment in PBMCs of AD patients showed that curcumin decreases the NF-κB, BACE1, and Toll-like receptor mRNA levels and upregulates the mRNA levels of mannosyl-glycoprotein 4-β-N-acetylglucosaminyltransferase (MGAT3) and the vitamin D receptor (VDR) [16,21,30]. BDC leads to diminished Aβ aggregates and inhibits proinflammatory induction [21,31]. Overall, these data support a protective effect of BDC. Furthermore, this latter effect has also been confirmed in in vivo studies. In fact, in an AD mouse model, BDC treatment suppressed the neuroinflammatory response [32]. Concerning Aβ degradation, BDC has been shown to have a relevant effect in the reduction of Aβ by the upregulation of Neprilysin, the most important Aβ-degrading enzyme [33]. In vivo experiments in the AD animal model showed a reduction of the Aβ content in mice fed with curcumin and BDC [34].

While oral curcumin is well-tolerated (turmeric curcumin is used as a spice in many cultures), the clinical utility of curcumin is limited because of its hydrophobicity, poor systemic bioavailability, and chemical instability [35]. To overcome these problems, many groups worked on developing new nanoformulations of curcumin to improve the delivery of the molecule and some improvement on the cognitive skills of treated subjects in some clinical trials [36,37].

In the current work, we decided to develop a nanoformulation of BDC to obtain a therapeutic agent able to regulate neuroinflammation and, ultimately, to benefit patients affected by AD. HFn nanoparticles were chosen as a drug delivery system (DDS) because of their potential to encapsulate different hydrophilic and hydrophobic drugs inside the inner cavity, thanks to their ability to modify the quaternary structure in response to pH changes [38].

HFn possess some unique features that are very appealing from the clinical point of view, as they are characterized by: (i) uniform architecture, (ii) negligible immunogenicity, and (iii) stability in the physiological environment [39]. Furthermore, HFn can cross the blood–brain barrier (BBB) by exploiting Transferrin receptor 1 (TfR1), which is overexpressed on the brain capillary endothelial cells [40]. Last, but not least, HFn protects the drug from a rapid degradation typical of curcuminoids [19].

In this work, we tested the solubility and stability of the BDC-HFn nanoparticles and then the effect of the BDC-HFn treatment on the transcriptomic profile of PBMCs from AD and healthy controls by RNA-seq. Furthermore, the ability of BDC-HFn to cross the blood–brain barrier (BBB) was assessed using an in vitro model.

## 2. Results

### 2.1. Hfn-BDC Nanoformulation

**Figure 1:**
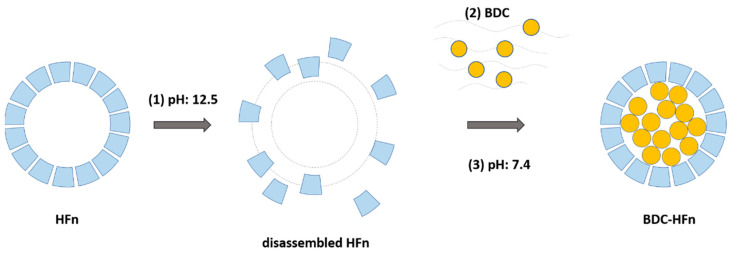
Schematic representation of the protocol used for the preparation of BDC-HFn.

HFn nanoparticles, consisting of 24 identical heavy-chain subunits of human heavy-chain Ferritin, were produced and purified, as previously reported [41]. For the encapsulation of BDC, the alkaline disassembly of the cages at high pH values was exploited (Figure 1). In this condition, HFn nanoparticles naturally lose their quaternary structure, and BDC increases its water solubility, avoiding a pre-solubilization step using organic solvents [39]. By quickly restoring the pH at neutral conditions, HFn reassemble as nanocages, and BDC remains trapped in the cavity, increasing its apparent solubility.

**Figure 2:**
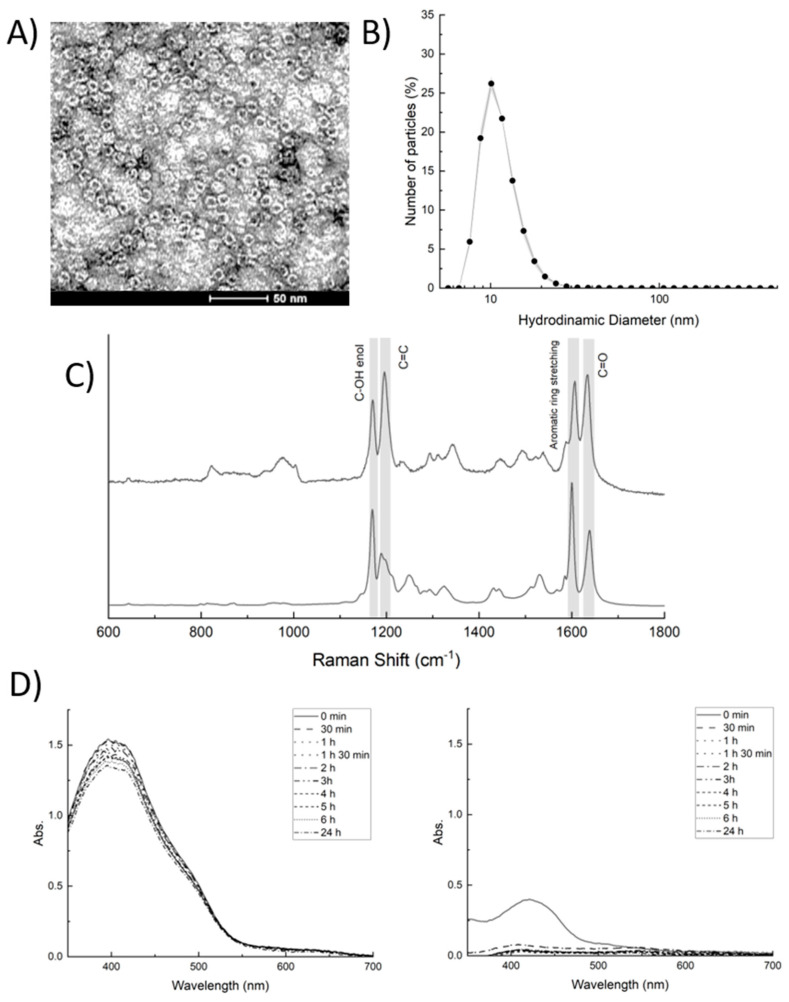
(**A**) TEM pictures of BDC-HFn nanoparticles. (**B**) DLS analysis of BDC-HFn nanoparticles. Data are presented as the mean of three independent acquisitions. (**C**) Raman spectra of BDC (top) and BDC-HFn (bottom). (**D**) UV–Vis spectra of BDC-HFn (left) and BDC (right) obtained at different time points between 0 min and 24 h.

The final BDC-HFn suspension was characterized from a morphological and dimensional point of view by TEM and DLS. Both analyses confirmed that the BDC encapsulation did not affect the structure of the nanocages, and the obtained BDC-loaded NPs were spherical, with a dynamic diameter of about 12 nm (Figure 2A,B). The comparison with the TEM analysis of HFn confirms that no major variation in the quaternary structure of the protein occurs during the loading (Figure S1).

In order to assess the efficient loading of BDC inside HFn nanocages, the encapsulated BDC molecules were extracted with acetic acid, exploiting the same disassembly strategy based on pH changes used for the encapsulation and quantified spectroscopically (Figure S2). The obtained data confirmed that, by using the proposed method, 27 ± 7 BDC molecules were encapsulated in each HFn, with a loading efficiency of 23.3 ± 8.3%. Furthermore, we performed a Raman spectroscopy analysis to confirm that BDC was not degraded during the encapsulation process. As shown in Figure 2C, the Raman spectrum of BDC-HFn shares all the prominent peaks observed in BDC conserving among the others, the peaks relative to the central α,β-unsaturated β-diketone moiety at 1168 and 1636 cm^−1^ [42,43], proving the structural integrity of BDC within HFn.

A stability test under physiological conditions was performed on free BDC and BDC-Hfn following the variations in the absorption profile over time. Free BDC had a typical absorption peak at 420 nm. As expected, this peak intensity dramatically decreased within 30 min, demonstrating that BDC is extremely poor water-soluble and has a low chemical stability under physiological conditions. On the contrary, BDC-HFn showed a slightly different profile with a maximal absorption peak at 405 nm, which remained stable for 24 h, maintaining its long-term integrity (Figure 2D).

### 2.2. RNA-Seq Differentially Expressed mRNAs and lncRNAs

Transcriptomic profiles of PBMCs isolated from AD patients and healthy controls (5 × 10^6^ cells with viability ≥ 80%) were studied at the basal condition (NT) and after a 24-h treatment with BDC-HFn (10 μM). No sign of cytotoxicity was detected after the treatment (Figure S3).

In this work, we analyzed five conditions:

1. AD NT vs. CTR NT;
2. AD BDC-HFn vs. CTR BDC-HFn;
3. AD NT vs. AD BDC-HFn;
4. AD BDC-HFn vs. CTR NT;
5. CTR NT vs. CTR BDC-HFn.

**Figure 3:**
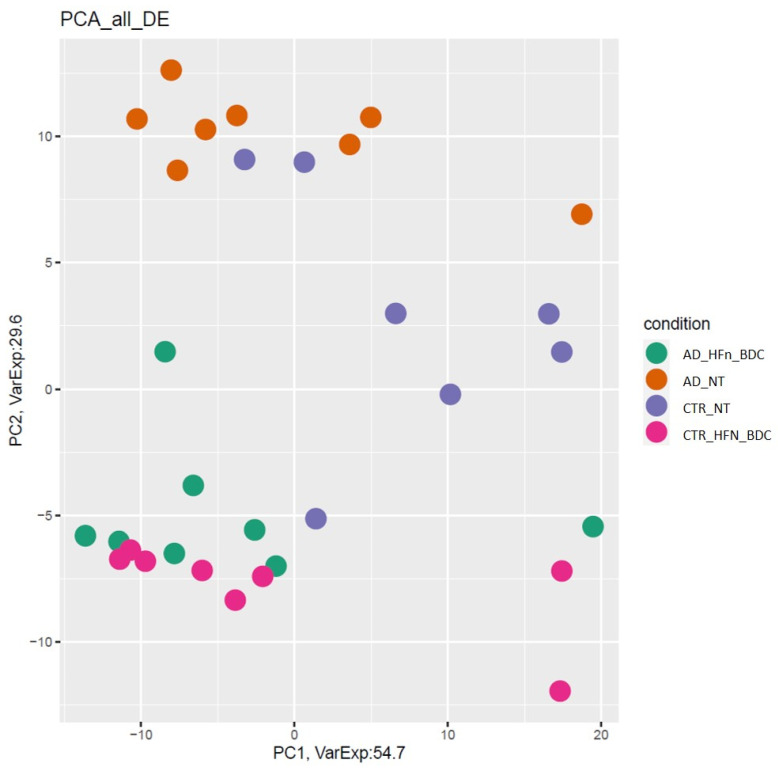
PCA of differentially expressed genes. All comparisons are given between the AD, AD BDC-HFn, and CTR BDC-HFn compared to CTR (AD_NT N° = 8, CTR_NT N° = 7, AD_Hfn_BDC N° = 8, and CTR_Hfn_BDC N° = 8).

Figure 3 showed a PCA of differentially expressed genes in all the analyzed conditions. CTR represents the separated group (in violet), and AD patients (in orange) are well-separated from other samples. Both AD and CTR-treated samples are mixed (green and fuchsia).

Various differentially expressed (DE) genes were found between patients and controls and before and after treatment. A summary of these results is represented in Table 1. The first evidence is relative to the number of DE transcripts among AD patients and controls. The total number of affected genes is 630, while no important difference has been shown after treatment of both patients and controls (98 DE genes). The most interesting data emerged from the comparison between AD before and after BDC-HFn treatment, where 2517 DEG were identified. Finally, the correlation between one group treated and one not treated (AD BDC-HFn vs. CTR and AD vs. CTR BDC-HFn) showed deregulation similar to AD vs. CTR in terms of the number of DEG genes. Next, mRNAs and lncRNAs were separated to understand the deregulation in both RNA groups. The list of all DE genes is reported in Supplementary Table S1.

#### 2.2.1. AD NT vs. CTR NT

**Figure 4:**
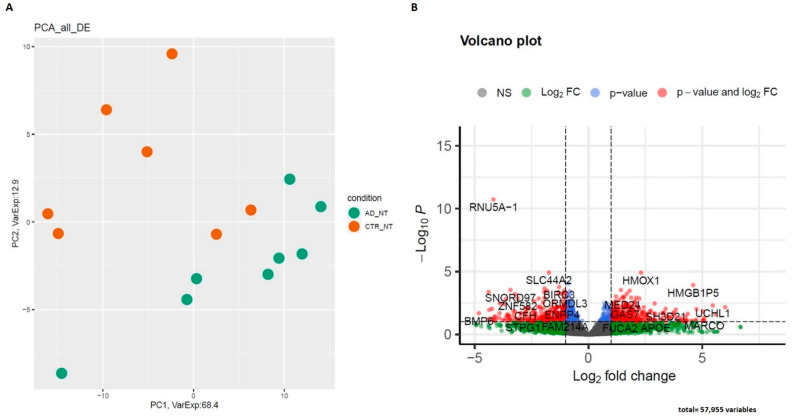
(**A**) PCA of differentially expressed genes. All comparisons are given between AD compared to CTR. (**B**) Volcano plot of differentially expressed genes between AD and CTR (AD_NT N° = 8 and CTR_NT N° = 7).

In AD patients, the RNA-seq data reported 630 DE genes: 63 were lncRNAs (11 upregulated and 52 downregulated genes), 8 out of 63 were reported as antisense, and 8 as lincRNAs, while the remaining 47 were classified as processed transcripts or intronic sense RNAs (Table S1). Concerning coding genes, 567 differentially expressed mRNAs were identified, 234 of which were downregulated, while 333 were upregulated. The PCA division based on the expression levels of all dysregulated mRNAs and lncRNAs in AD and CTR is represented in Figure 4.

The most deregulated coding genes were CDCA2, SLC1A2, and CXCL5 (downregulated) and UCHL1 and MARCO (upregulated). Interesting, CXCL5 and MARCO are both related to inflammation [44,45], while UCHL1 is a gene specific for Parkinson’s disease but also described in AD [46].

The most deregulated genes associated with inflammation are represented in Table 2. Regarding lncRNAs, the most DE are CTA-445C9.14, described as TFIP11-AS, and AC114271.2 and ICAM3-AS, an intercellular adhesion molecule already associated with AD [47].

#### 2.2.2. AD NT vs. AD BDC-HFn

**Figure 5:**
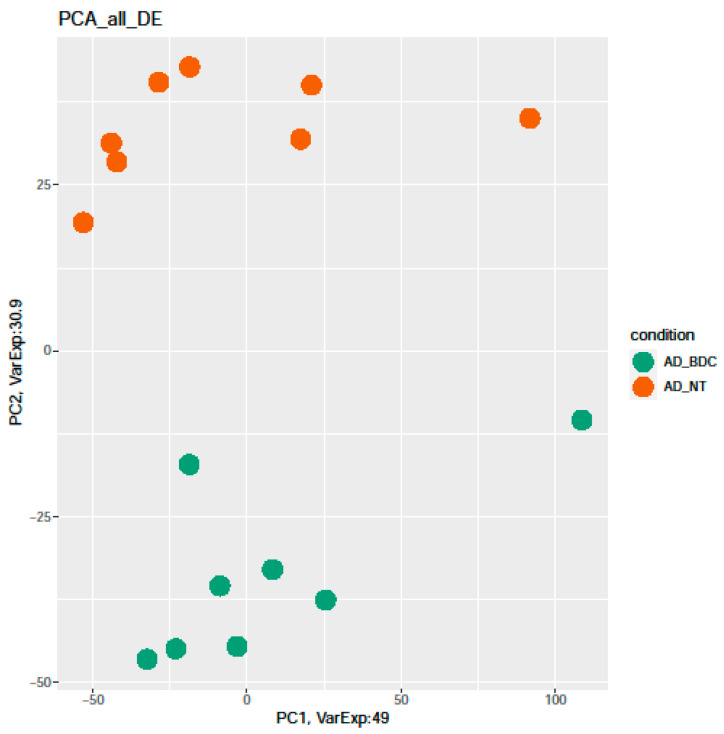
PCA of differentially expressed genes. All comparisons are given between AD untreated compared with AD after BDC-HFn treatment (BDC N° = 8 and CTR_Hfn_BDC N° = 8).

In AD patients, BDC-HFn treatment showed a significant increase in DE genes detected (2517) (Table S2). Genes upregulated were 1419 and 88 lncRNAs, while 952 coding and 50 lncRNAs were downregulated. Thirty-eight of the total number of lncRNAs were classified as antisense. As already mentioned, the genes most deregulated in AD after treatment were linked to inflammation, such as CXCL10, CXCL9, and CCL8, which were downregulated, and the interleukins, which were upregulated. Moreover, after BDC-HFn treatment, the APOE gene was also downregulated compared to the untreated patients. The PCA division based on the expression levels of all dysregulated mRNAs and lncRNAs in AD before and after treatment is represented in Figure 5.

**Figure 6:**
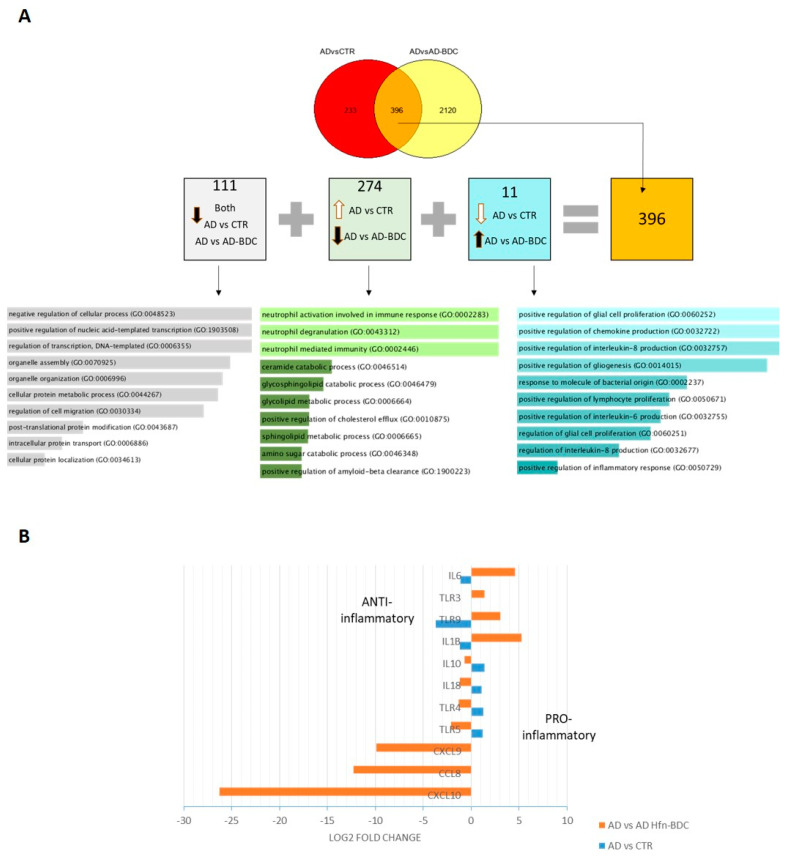
Comparison between DEG genes obtained by AD_NT vs. CTR_NT and AD_NT vs. AD_Hfn_BDC. (**A**) Venn diagram of DEG genes in AD before and after treatment, and its distribution in terms of the deregulation sign of 396 common genes found. Lists of genes are reported in Table S6. For each group, GO biological process analysis have been reported. (**B**) Graphical representation of the main genes that change with AD BDC-HFn treatment.

Moreover, the analysis of the AD NT vs. AD BDC-HFn list of DEG genes showed that 396 genes were DEG in both conditions (Figure 6A). Next, we investigated the trend of deregulation in each condition. About one-third (111 genes out of 396) were downregulated in untreated and treated PBMCs from AD patients, 274 were upregulated in the AD_NT samples while downregulated after Hfn-BDc treatment, and 11 genes were downregulated at the basal condition and changed to upregulation after Hfn-BDC treatment (Figure 6A).

In detail, it seems that the genes that belong to the immune system are responsive to Hfn-BDc treatment. For example, the expression of some genes of the CXLCL family that were downregulated in AD compared to the controls after BDC-HFn treatment was found to be upregulated. Additionally, the IL family (as IL1 and 6) decreased expression after BDC-HFn, while they were upregulated in the AD not treated (Figure 6B).

The data obtained from the comparison of AD BDC-HFn vs. CTR BDC-HFn, AD BDC-HFn vs. CTR NT, and CTR NT vs. CTR BDC-HFn are reported in the Supplementary Files (Figures S4 and S5 and Tables S3–S5).

### 2.3. Validation of Deregulated Coding and Noncoding Genes

To confirm the RNA-seq results, we performed qPCR for a subset of selected mRNAs and lncRNAs. We preferably chose the transcripts to be validated among the most differentially expressed found; for this reason, we validated MARCO (Macrophage Receptor with Collagenous Structure), CDK3 (Cyclin-Dependent Kinase 3), GSTM1 (Glutathione S-Transferase Mu 1), and CDCA2 (Cell Division Cycle-Associated 2) (Figure S6).

### 2.4. mRNA Pathway Analysis

#### 2.4.1. AD NT vs. CTR NT

**Figure 7:**
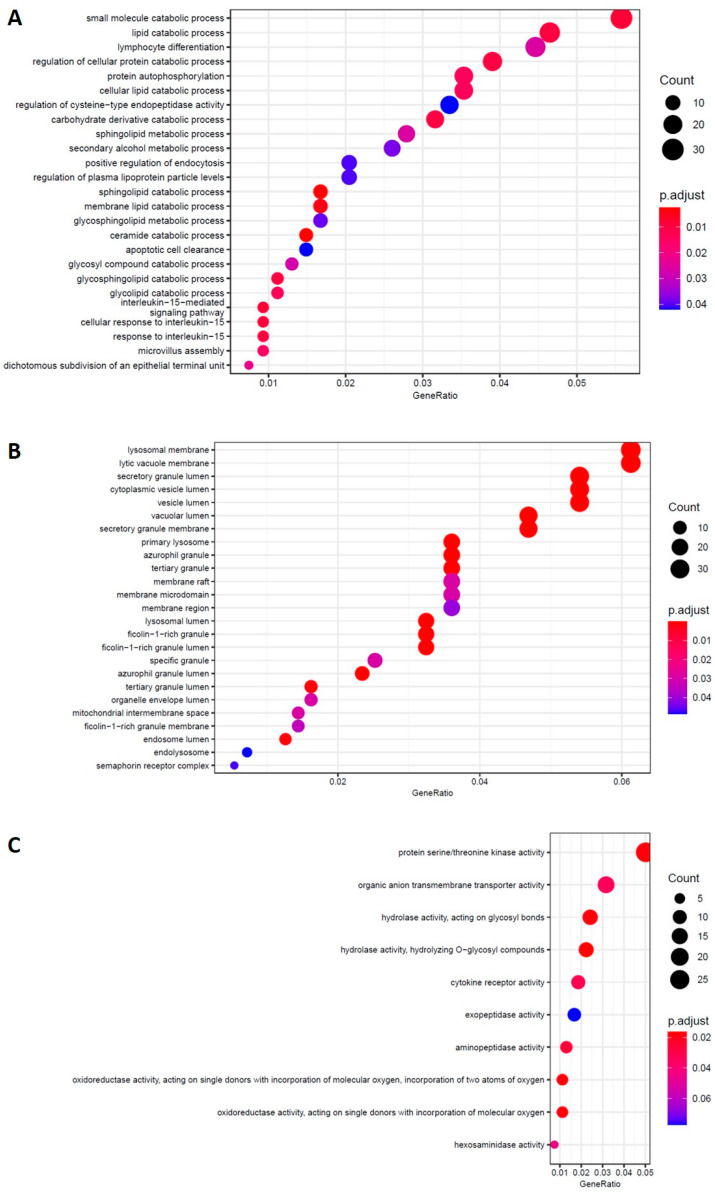
AD vs. CTR. GO-enriched terms for the biological process (**A**), cellular component (**B**), and molecular function (**C**).

Gene ontology (GO) terms enrichment analysis and the KEGG pathways for DEs in untreated PBMCs from AD patients compared to healthy controls were run [55] (Figure 7). The GO biological process analysis resulted in apoptotic signaling and interleukin pathways. Enriched GO terms for cellular components concern the lysosome membrane and vesicle lumen. In the list of affected pathways obtained through the KEGG from up- and downregulated genes; it also reported “Cytokine-cytokine receptor interaction, lysosome and NF-kappa B signaling pathway” as some of them.

#### 2.4.2. AD NT vs. AD Hfn-BDC

**Figure 8:**
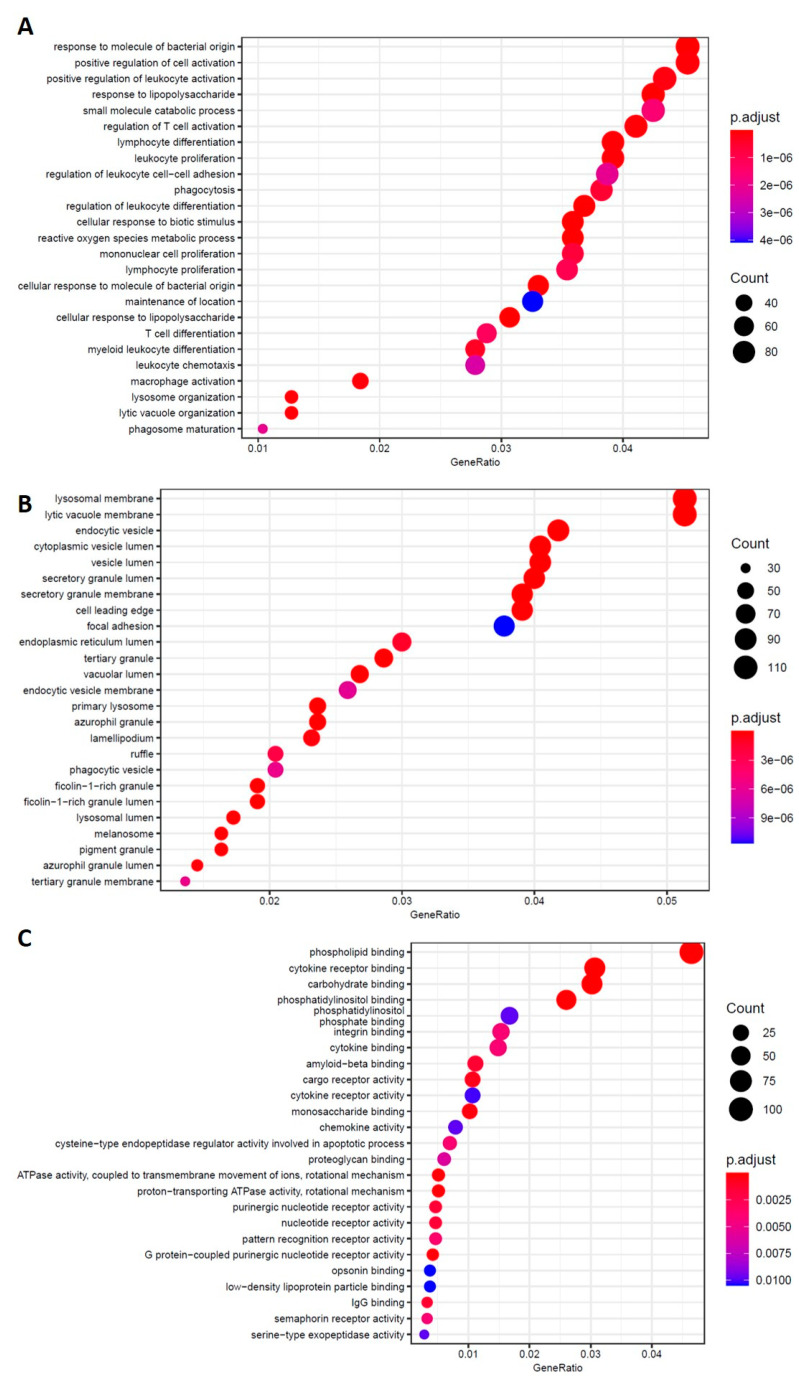
PBMCs isolated from AD patients untreated vs. treated with Hfn-BDC. GO-enriched terms for the biological process (**A**), cellular component (**B**), and molecular function (**C**).

Concerning the biological processes and molecular function pathways analyses, we detected an important involvement of the immune system, such as macrophage and leukocyte activation and cytokine binding. Related to these pathways, the macrophage and chemokine receptors and interleukins genes were altered. Moreover, the phagocytosis and amyloid beta binding pathways were also found altered. Enriched GO terms for the cellular component were the lysosome membrane and vesicle lumen (Figure 8), as observed in untreated AD vs. the CTR (Figure 7).

#### 2.4.3. AD BDC-HFn vs. CTR BDC-HFn

A pathway analysis investigation of the biological process showed an involvement of the negative regulation of locomotion and movement, cells migration, and chemotaxis (Figure S7); while regarding the molecular function, the most interesting data concern GTPase activity, which was previously demonstrated to be altered in AD [56]. This comparison did not show an innovative pathway due to the small number of DEG.

#### 2.4.4. AD BDC-HFn vs. CTR NT and CTR NT vs. CTR BDC-HFn

These two comparisons showed similar deregulated pathways with respect to other conditions. In particular, the evaluation between AD BDC-HFn and CTR showed a difference in terms of the cytokines, leukocytes, and macrophages. GO enrichment of CTR untreated vs. treated showed the alteration of the pathways that may be linked to the immune response (Figure S8).

### 2.5. BDC-HFn Penetration across a BBB In Vitro Model

Many research studies demonstrated that HFn is a suitable nanoparticle to transport drugs across the BBB, because it specifically binds the TfR1 receptor, found overexpressed on the surface of the BBB endothelium [40,57,58]. Based on this observation, we investigated the ability of BDC-HFn to cross an in vitro model of BBB made of immortalized murine endothelial cells of the cerebral cortex (bEnd.3) seeded in a transwell system.

**Figure 9:**
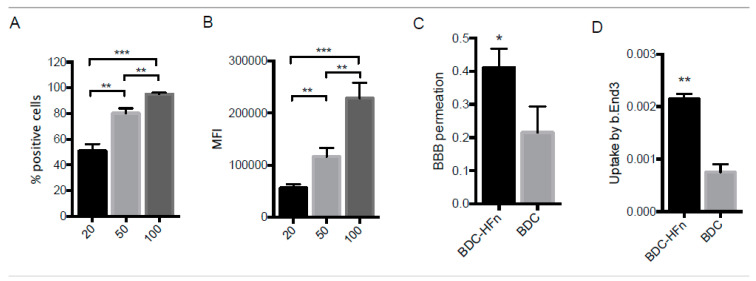
(**A**) Dose-dependent HFn binding to b.End3 endothelial cells was assessed by flow cytometry using 20, 50, and 100 µg mL^−1^ of FITC-labeled HFn. (**B**) Mean fluorescence intensity (MFI) of HFn-bound b.End3 cells. (**C**) Permeation of BDC-HFn or free BDC through an in vitro BBB model was calculated as the percentage of BDC collected in the lower compartment of treated transwells as compared to the incubated dose. (**D**) Percentage of uptake of BDC-HFn or free BDC by bEnd.3 cells. Reported values are the means of 3 replicates ± std. dev. * *p* < 0.05, ** *p* < 0.01, and *** *p* < 0.001 (unpaired Student’s *t*-test).

Before performing the assay, dye-labeled HFn was incubated with bEnd.3 cells to assess the HFn-TfR1 interaction. By using increasing concentrations of HFn (20, 50, and 100 µg mL^−1^), we confirmed a dose-dependent binding of HFn on the cells, as demonstrated by increasing the percentage of stained cells and augmented fluorescence intensity (Figure 9A,B).

Then, we established an in vitro BBB model by seeding bEnd.3 cells on the apical side of the transwells and let them grow for 10 days. The formation of tight junctions in the cellular monolayer was checked by measuring the TEER and the permeability of a fluorescently labeled tracer of high molecular weight (Figure S9). After 10 days of culture, the recorded mean TEER value was 53.3 ± 2.5 Ω × cm^2^, consistent with the TEER of monoculture models reported in other research studies [59,60]. The permeability of FD40 across the endothelial layer (4.84 × 10^−7^ cm sec^−1^) supported the low paracellular flux of molecules and integrity of the BBB model. BDC-HFn (100 µg mL^−1^) and an equivalent dose of free BDC were incubated in the transwell apical compartment diluted in the culture medium. After 3 h of incubation, the amount of BDC collected in the transwell lower compartment was measured by mass spectrometry. The percentage of endothelial permeability revealed that BDC-HFn nanoformulation enhanced BBB permeation by almost two-fold as compared to free BDC (*p* = 0.01, Figure 9C). We also evaluated the drug uptake in BBB endothelial cells to prove nanodrug transcytosis. The results showed an increased internalization of BDC-HFn compared to free BDC (*p* = 0.0003, Figure 9D), thus confirming that the nanoformulation was able to mediate the in vitro trans-BBB delivery of BDC through a transcellular passage. By contrast, free BDC, poorly water-soluble and unstable, showed the inability to undergo physiological uptake by BBB endothelial cells. In order to rule out any potential bias due to alterations of the cell viability induced by the nanoparticles, endothelial cells were incubated with BDC-HFn, and the viability was assessed after 24 h. No cytotoxic effect was observed upon treatment with increasing nanoparticle concentrations (Figure S10), confirming that BDC-HFn does not impact the survival of BBB cells but, rather, enhances drug transportation through live cells.

## 3. Discussion

The use of HFn nanoparticles for BDC formulation allowed us to obtain a water-soluble, easy to handle, and stable drug that can be effectively used to treat the PBMCs from AD patients and healthy controls (HC). In addition, the applied loading protocol did not require an additional surfactant or organic solvents, limiting the procedure’s impact on HFn and reducing the risk of contamination. PBMCs are a good preliminary model of disease, because they share the same mechanisms that are deregulated in CNS [8], but they are much more easily accessible. The next step is to understand whether BDC-HFn may be an interesting therapeutic solution in “in vivo” validation.

The data obtained from the characterization of BDC-HFn confirmed that the developed nanoparticles are small, with a measured hydrodynamic diameter of about 12 nm. This value perfectly matches the literature reported for HFn nanocages in physiologic conditions. Additionally, TEM images of BDC-HFn confirmed the characteristic cage shape of HFn, suggesting that the protein’s secondary and tertiary structures are not affected by the presence of BDC. Raman spectroscopy is a vibrational spectroscopy that provides fingerprint information on the chemical groups present in the sample analyzed that is vastly adopted in the analysis of pharmaceuticals [61]. One of the strengths of Raman is its ability to work on virtually any kind of molecule or formulation, and that requires a minimal amount of samples to be performed. The results obtained confirmed the presence of the characteristic chemical groups present in BDC. However, a minor shift in the position or intensity of the peaks can be detected, suggesting a conformational change in the drug once encapsulated within HFn. More in detail, the difference in the relative intensity between the peaks at 1170 and 1196 cm^−1^ relative to the groups involved in the cheto-enolic ring at the center of the molecule suggests that BDC in HFn nanoparticles assumes a more planar conformation [62].

The obtained data also prove that BDC-HFn are stable in water at room temperature and do not show any sign of degradation for 24 h, thus drastically improving the drug benefit.

Concerning transcriptomic data, the first relevant data concerned the different RNA profiles between the AD patients and healthy controls that showed substantial deregulation related to AD disease (630 DEG). Interestingly, numerous deregulated genes are related to inflammation and immune-response pathways, such as the CXCL chemokine family, interleukins, and TLR [63,64]. In fact, the gene ontology analysis showed the involvement of the response to the interleukin and lymphocyte differentiation pathways. Moreover, the AD pathways and genes specific to the clearance of Aβ and APOE expression have been found [65]. Indeed, between DE genes, APOE expression has been found altered.

Regarding lncRNAs, the most interesting data concern two lncRNAs-AS, IFITM3-AS and ICAM3-AS; both sense genes have already been demonstrated to be associated with AD [47,66]. The first is interferon-induced transmembrane protein, while ICAM3 is a cell adhesion molecule.

A significant increase in deregulated genes was noted when we compared AD patients treated with BDC-HFn and AD not treated (2517). Here, we observed that a group of genes related to inflammation changed in terms of expression.

By the analysis of common genes detected in AD_NT vs. CTR_NT and AD_NT vs. AD_Hfn_BDC conditions, we identified two groups of genes that convert from up- to downregulated (274) and down- to up- (11) after treatment. About genes that increased their expression after HFn-BDc treatment, three of the biological processes involved concern neutrophils activation that has been already demonstrated; indeed, the proteins related to neutrophils activation are upregulated [67]. After HFn-BDC treatment, the genes associated with this pathway were decreased, such as TIMP2 [68]. Concerning genes that increased their expression after Hfn-BDC treatment, the GO results showed a main positive regulation of interleukin, glia cells, and chemokines.

In fact, proinflammatory genes such as CXCL9 and 10 and interleukins such as IL10 and IL18 decreased after BDC-HFn treatment. In contrast, anti-inflammatory genes such as IL6, IL1B, and TLR9 have been found to be upregulated in response to BDC-HFn treatment.

These data suggest a specific anti-inflammatory effect of BDC-HFn in PBMCs from AD patients by improving the anti-inflammatory genes and inhibiting the proinflammatory genes. The pathway analysis confirms this trend, as in the list of the involved pathways after BDC-HFn treatment, there are the interleukin signaling, cellular response, and cytokines response activity. However, regarding AD BDC-HFn vs. CTR BDC-HFn, the comparison did not show an innovative pathway due to the small number of DEG and none AD-specific, even if the BDC-HFn treatment in both patients and controls did not highlight the disease mechanism in terms of the gene expression.

The last two correlations, AD BDC-HFn vs. CTR NT and CTR NT vs. CTR BDC-HFn, showed similar deregulated pathways to other conditions. In particular, the evaluation between AD BDC-HFn and CTR showed differences in terms of cytokines, leukocytes, and macrophages and pathways that are deeply altered by curcuminoids [69].

Our results also confirmed that HFn nanoformulation can bind brain endothelial cells in a dose-dependent manner and favors the trans-BBB crossing of intact BDC through an established BBB model. Furthermore, the cellular uptake experiment revealed an increased internalization of BDC-HFn in b.End3 culture compared to free BDC, and no nanoparticle-mediated cytotoxic effect was observed in these cells. Taken together, these data demonstrate the capability of BDC-HFn to enhance BBB permeation in vitro and provide preliminary evidence suggesting that HFn may deliver BDC across the BBB endothelium to reach the brain, where the anti-inflammatory activity is mainly required to achieve neuroprotection.

It should be noted that, for the drug permeability assessment, we used a transwell system that, although accepted and globally used, presents some limitations [70,71]. First, it comprises a monolayer of brain endothelial cells and lacks supportive glial cells, making it a rather simplistic model [72]. Second, it is a static system, while, in vivo, a continuous dynamic blood flow may alter the drug interaction with the BBB endothelium [73]. Finally, there will always be the question of how accurate the 2D morphology of in vitro transwell represents cerebral vessels’ in vivo situations [74].

Nevertheless, the model we chose has several advantages: ease to handle, low cost, rapidity, and experimental reproducibility [75,76]. Therefore, for our study, we reasoned that it would have been suitable to gain insights of our novel nanoformulation capability to overcome or not the BBB barrier. Further studies will be required to correlate the in vitro permeability data obtained in other more impermeable models and increase the overall predictability of our results.

Overall, our data showed how BDC-HFn could improve the pharmacokinetic properties of the drug and that significant differences are present in the gene expression between the same patients before and after BDC-HFn treatment, particularly in genes associated with inflammation. Moreover, inflammatory genes that are upregulated between AD and CTR after BDC-HFn treatment are converted into those downregulated, supporting a possible therapeutic approach.

## 4. Materials and Methods

### 4.1. Nanoformulation and Characterization of BDC-HFn

#### 4.1.1. HFn Loading with Bisdemethoxycurcumin (BDC)

The loading of BDC within the central cavity of HFn nanoparticles was achieved following a protocol based on the disassembly/reassembly of the protein cage at basic pH previously published for curcumin (Cur) and described in [77]. Briefly, the pH of an HFn solution (1 mg/mL in 0.15 M NaCl) was adjusted to 12.5 using an appropriate volume of NaOH (1 M). The solution was then left at room temperature for 15 min to allow the disassembly of the nanocage structure. After that, a solution of BDC freshly solubilized in NaOH (0.1 M) was added, and the pH was immediately brought back to neutrality. The resulting solution of the protein and BDC was then stirred in the dark at room temperature for 2 h to promote the refolding of the quaternary structure of HFn. The obtained product was centrifuged at 10,000 rcf for 10 min to remove the excess of insoluble BDC, which made the solution turbid. The final solution was washed several times with sterile PBS buffer and concentrated using a 100-kDa Amicon centrifuge filter (Millipore Corporate, Merck KGaA, Darmstadt, Germany). At last, BDC-HFn nanoparticles were purified using a Zeba Spin Desalting Column (Thermo Scientific, Monza, Italy) to remove the excess free drug, eventually remaining in the solution and the adsorbed molecules.

#### 4.1.2. BDC-HFn Nanoparticles Characterization by Transmission Electron Microscope and Dynamic Light Scattering

For the TEM analysis, a small drop of BDC-HFn solution was deposited and dried over a Formvar-coated copper grid at room temperature. The sample was stained with uranyl-acetate 1% for 30 s at room temperature and dried overnight. The samples were evaluated by TEM (Tecnai Spirit, FEI, Hillsboro, OR, USA) at 300,000× and 80,000× magnifications. A dynamic light scattering (DLS) analysis was performed by suspending BDC-HFn nanoparticles in phosphate buffer at a pH 7.2 at a final concentration of 1 mg/mL of HFn.

#### 4.1.3. Determination of Drug Loading Efficiency and Stability of BDC-HFn

A spectrophotometric approach was used to determine the efficiency of BDC loading within HFn nanocages. First, a calibration line was obtained by measuring the spectra of standard solutions of BDC (from 0 to 100 μM) dissolved in acetic acid immediately after preparation. The efficacy of the loading was determined by comparing the intensity of the absorbance peak at 415 nm of the solution of BDC-HFn with a calibration curve using the EnSight Multimode Plate Reader (PerkinElmer) (Figure S2).

The stability of BDC-HFn was measured under physiologic conditions at 37 °C and compared with the one of the free BDC. BDC-HFn was suspended in PBS at pH = 7.2 at a 50-μM final concentration. Free BDC was dissolved in DMSO at 50 mM and then diluted in PBS buffer for the stability experiments at 100 μM. The final concentration of DMSO in the solution was negligible. A 2-mL aliquot of each solution was transferred into a cuvette at different time points (0 min, 30 min, 1, 2, 3, 4, 5, 6, and 24 h), and the absorption spectrum was measured using a NanoDrop 2000c UV−Vis spectrophotometer (Thermo Scientific).

#### 4.1.4. Raman Spectroscopy

Raman spectra were recorded using an InVia Reflex confocal Raman microscope (Renishaw, Wootton-under-Edge, UK) equipped with 3 laser light sources operating at 533, 633, and 785 nm. The Raman spectrometer was calibrated daily using a silicon wafer using a peak at 520.7 cm^−1^. The spectra of BDC and of BDC-HFn were acquired from a small amount of sample deposited and dried at room temperature on a CaF_2_ slide (Crystran, Poole, UK) without any further preparation. The Raman spectra were acquired using a 785 nm laser, 1200-L/mm grating, and a 100× objective collecting signal for 10 s.

### 4.2. Blood Brain Barrier Model (BBB)

Mouse brain endothelial cells (bEnd.3) were kindly provided by Dr. Pompa (Italian Institute of Technology, Genova, Italy) and cultured in high-glucose DMEM medium supplemented with 10% FBS, 1% nonessential amino acids, 1% L-glutamine, and 1% penicillin/streptomycin. The cells grew at 37 °C in humidified atmosphere containing 5% CO_2_ and were subcultured prior to confluence using the trypsin/EDTA solution. The cell culture medium and chemicals were purchased from EuroClone S.p.A, (Pero, Italy).

To set up a BBB model, cells (2 × 10^5^) were seeded in the upper chamber of the transwell inserts (Corning^®^ polyester membrane cell culture inserts, pore size 0.4 µm, 4.2 cm^2^ area) and let to adhere overnight. The medium was changed the day after seeding and then every two days by putting 1 mL in the upper chamber and 2 mL in the lower compartments. Four transwells were left without cells and used as the blank control. To assess the formation of a tight monolayer, the transepithelial electrical resistance (TEER) was measured four days after cell seeding using an electrode device (EVOM2, World Precision Instruments). For each transwell, the mean TEER value of the blank inserts was subtracted from the TEER value of the BBB, and the results were expressed as Ω × cm^2^. The measurements were repeated every two days.

For BBB validation, 1 mg mL^−1^ FITC-dextran (FD40, molecular weight 40,000, Sigma-Aldrich) was added to the upper compartment of three BBB models and three empty inserts. After 1, 2, and 3 h, 200 μL were withdrawn from the lower chamber, and the fluorescence intensity was measured by spectrofluorometer (λ_ex_ 488 nm and λ_em_ 515 nm). The amount of permeated FD40 was determined by comparison of the observed fluorescence values with a calibration curve produced with known concentrations of the fluorescent tracer dissolved in the culture medium. The permeability coefficient was calculated according to the following equation: Papp = (basolateral concentration * basolateral volume)/(incubation time * membrane area * initial concentration in the apical side). The percentage of transcytosed FD40 was calculated as: Final basolateral concentration/Expected Concentration at equilibrium.

For the transport study, on day 10 after cell seeding, the BBB upper compartments were incubated with 100 µg mL^−1^ of BDC-HFn or the corresponding concentration of free BDC in the culture medium for 3 h to test the permeation of the nanocage through the BBB. Two BBB inserts were left untreated and were used as blank controls. At the end of the incubation, all media in the upper and lower chambers were collected and stored at −20 °C until UPLC/MS-MS measurements of the BDC content. After two washes with PBS, the cells were detached from the insert and centrifuged 5 min at 500× *g*. The cell pellets were washed with 1 mL of PBS and stored at −80 °C for analysis of the drug uptake by UPLC/MS-MS. 

### 4.3. Cell Binding Assay

For the cell binding experiment, HFn was labeled with fluoresceine isothiocyanate Isomer I (FITC, Sigma-Aldrich S.r.l., Milan, Italy, CAS Number: 3326-32-7), according to the manufacturer’s protocol. Endothelial cells (5 × 10^5^) were incubated for 2 h at 4 °C in flow cytometry tubes in the presence of 20, 50, or 100 μg mL^−^^1^ of FITC-labeled HFn diluted in PBS–0.3% bovine serum albumin (BSA, Sigma-Aldrich S.r.l.). After incubation, cells were washed three times with PBS, resuspended with 0.5 mL of phosphate-buffered saline (PBS, EuroClone), and analyzed by a CytoFLEX flow cytometer (Beckman Coulter, Cassina De Pecchi, Italy). A total of 20,000 events were analyzed for each sample after gating on viable cells and on singlets. The appropriate gates were set using a sample of untreated cells.

### 4.4. Endothelial Cells Viability Assay

bEnd.3 cells (5 × 10^3^ cells/well) were seeded in a 96-well plate and treated with 20, 50, or 100 μg mL^−^^1^ of BDC-HFn in a cell culture medium for 24 h. At the end of the incubation, the cells were washed with PBS and incubated with 20 μL of MTS reagent (CellTiter 96^®^AQueous One Solution Cell Proliferation Assay, Promega) diluted into 80 μL of phenol red-free DMEM for 3 h at 37 °C. The absorbance was read at 490 nm and a reference wavelength of 620 nm. Untreated samples were used to normalize the results and set them at 100% viability.

### 4.5. BDC Measurement by UPLC/MS-MS

BDC determination in LC samples was performed by UPLC–tandem mass spectrometry (MS/MS) in detail with Waters Acquity UPLC-TQD.

The MS/MS conditions were as follows: interface, electrospray in the positive ion mode; multiple reaction monitoring acquisition, *m*/*z* 308.92 → 146.95 (CV 32, CE 25) for BDCur; *m*/*z* 375.2 → 180.04 (CV 30, CE 21) for the internal standard, curcumin D6.

The chromatographic conditions were as follows: column, Waters Acquity UPLC BEH Shield RP18 (1.7 μm, 2.1 × 100 mm) maintained at 45 °C; eluent A, 0.1% formic acid in water; eluent B, acetonitrile; flow rate, 0.45 mL min^−1^. A gradient program was employed for chromatographic separation with solvent A and B as follows:

52%B (0–0.3 min), 52–75% B (0.3–3.0 min), 75–99% B (3.0–3.1), 99%B (3.1–5.5 min), and 99–22%B (5.5–6.0 min), followed by 2 min of re-equilibration time. The retention time of enol and keto BD Cur were 1.81 and 1.94 min, respectively; the retention time of curcumin D6 was 1.7 min.

All the volume (1.4 mL) obtained from the lower chamber underwent liquid–liquid extraction with methyl tert-butyl ether (5 mL × 2 in 20 + 20 min and using a vortex); the solvent was evaporated with nitrogen and then reconstituted with 400 μL of methanol before injection in UPLC (3 μL). The calibration curve was prepared in medium in the BDC concentration range of 1–20 μg/L. The detection limit (signal-to-noise ratio = 3) was 0.1 μg/L. The quantification of BDC is the sum from the enol and keto form.

### 4.6. Study Subjects

Fifteen AD patients and fifteen age- and sex-matched healthy controls (CTR) were recruited after obtaining written informed consent (Table 3). All the subjects were deep-sequenced and included in real-time PCR experiments. The AD patients underwent clinical and neurologic examination at IRCCS Mondino Foundation (Pavia, Italy). An AD diagnosis was made according to the National Institute of Neurological and Communicative Disorders and Stroke and the AD and Related Disorders Association (NINCDS-ADRDA) criteria [78]. The control subjects were recruited at the Transfusional Service and Centre of Transplantation Immunology, Foundation San Matteo, IRCCS (Pavia, Italy). The study protocol to obtain PBMCs from the patients and controls was approved by the Ethical Committee of the IRCCS Mondino Foundation (Pavia, Italy). Before being enrolled, the subjects participating in the study signed an informed consent form (Protocol no. 20200042334—version 18 May 2020). All experiments were performed in accordance with the relevant guidelines and regulations.

### 4.7. PBMCs Isolation, Treatment, and RNA Extraction

Peripheral blood mononuclear cells were prepared by layering peripheral blood on Ficoll–Histopaque (density = 1.077) and centrifugation at 950× *g* for 30 min. After isolation on a Ficoll–Histopaque layer (Sigma, Italy), the cell viability was assayed by a trypan blue exclusion test and by a cytometric analysis [79,80]. Viable cells were used for in vitro studies with curcumins. PBMCs isolated from AD patients and healthy controls (5 × 10^6^ cells with viability ≥ 80%) were cultured for 24 h both at the basal conditions (NT) and treated for 24 h with BDC-HFn (10 μM). The experiment is composed by 4 different conditions:

- PBMCs isolated from AD patients not treated (AD NT);
- PBMCs isolated from AD patients treated with BDC-HFn (AD BDC-HFn);
- PBMCs isolated from Controls not treated (CTR NT);
- PBMCs isolated from controls treated with BDC-HFn (CTR BDC-HFn).

After treatment, the cell viability was assayed by a trypan blue exclusion test and confirmed using a flowcytometric analysis with a Zombie dye. The total RNA was isolated by the Trizol reagent (Life Science Technologies, Waltham, MA, USA), according to the manufacturer’s instructions. Quantification and quality control of the RNAs was done using a Nanodrop ND-100 Spectrophotometer (Nanodrop Technologies, Wilmington, DE, USA) and a 2100 Bioanalyzer (Agilent RNA 6000 Nano Kit, Waldbronn, Germany). RNAs with a 260:280 ratio of ≥1.5 and an RNA integrity number of ≥8 were selected for deep sequencing.

### 4.8. Libraries Preparation for RNA-Seq and Bioinformatic Data Analysis

Sequencing libraries of the AD patients and matched controls were prepared with the Illumina TruSeq Stranded RNA Library Prep kit, version 2, Protocol D, using 500 ng total RNA (AD_NT N° = 8, CTR_NT N° = 7, AD_Hfn_BDC N° = 8, and CTR_Hfn_BDC N° = 8). The quality of the sequencing libraries was assessed by the 2100 Bioanalyzer with a DNA1000 assay and DNA High-Sensitivity assay. RNA processing was carried out using Illumina NextSeq 500 Sequencing. FastQ files were generated via lllumina bcl2fastq2 (Version 2.17.1.14—http://support.illumina.com/downloads/bcl-2fastq-conversion-software-v217.html (accessed on 14 January 2021)) starting from raw sequencing reads produced by an Illumina NextSeq sequencer. Gene and transcript intensities were computed using STAR/RSEM software (1.3.3), Madison, WI, USA [81], using Gencode Release 19 (GRCh37.p13) as a reference and using the “stranded” option. A differential expression analysis for mRNA was performed using R package EBSeq [82]. This tool was selected because of its superior performance in identifying isoform differential expressions [83]. A differential expression analysis for long noncoding RNAs was performed with the R package DESeq.2 [84]. Coding and noncoding genes were considered differentially expressed and retained for further analysis with |log2(disease sample/healthy control)| ≥ 1 and an FDR ≤ 0.1. We imposed a minimum |Log2FC| of 1 and an FDR lower than 0.1 as the thresholds to differentially expressed genes. This choice is motivated by the decision to maximize the sensitivity of this analysis in order to perform a massive screening and identify candidate genes to be validated with real-time analysis. The RNA sequencing data are available in the GEO repository (GS).

### 4.9. Pathway Analysis

A gene enrichment analysis was performed on the coding genes [55]. We performed a gene ontology (GO) analysis for the biological processes, cellular components, and molecular function and a KEGG pathway analysis (Kyoto Encyclopedia of Genes and Genomes: http://www.genome.ad.jp/kegg (accessed on 1 February 2021)) via the enrichR web tool [85,86].

### 4.10. Real-Time PCR

AD_NT N° = 15 and CTR_NT N° = 15 were used for validation. The PCR oligonucleotide for sense genes pairs was selected spanning introns to optimize the amplification from mRNA templates and avoid nonspecific amplification products using NCBI’s Primer-BLAST or online Primer 3.0. Moreover, the primers were designed in specific regions that do not overlap with antisense sequences (primers upon request). The total cDNAs were prepared using the iScript™ cDNA Synthesis Kit (Bio-Rad, Richmond, CA, USA). Real-time PCR (qPCR) reactions were performed with SYBR Green SuperMix (Bio-Rad, Richmond, CA, USA) using 1 μL of cDNA template (or water control). The cycle threshold (Ct) values were normalized against those determined for GAPDH. Fold expression differences relative to the healthy controls were determined using the 2ΔΔCt method. The significance of the gene expression changes relative to the controls was analyzed using one-way ANOVA (Kruskal–Wallis) and Dunn’s posttest for all possible test pairings using Prism GraphPad 5.02 software (GraphPad Software, San Diego, CA, USA).

## 5. Conclusions

In summary, the present work describes the preparation of a new nanoformulation of bisdemethoxycurcumin within H-Ferritin nanocages (BDC-HFn) that can be prepared using a simple and very gentle approach that does not require any cosurfactants of additional organic solvents. The obtained BDC-HFn nanoparticles can enhance the stability of the drug in water and increase the delivery of BDC across the BBB in an in vitro model. Furthermore, BDC-HFn has a strong anti-inflammatory effect on PBMC obtained from Alzheimer’s disease patients. Overall, these data support the further development of BDC-HFn as a potential therapy for AD, which efficacy must now be confirmed in vivo.

## Figures and Tables

**Table 1:** Statistically significant differentially expressed RNA numbers in PBMCs from AD and controls untreated and treated in terms of upregulated transcripts, downregulated transcripts, and the total.

	Upregulated		Downregulated		Total RNAs
	mRNA	lncRNA	mRNA	lncRNA	
AD NT vs. CTR NT	333	11	234	52	630
AD NT vs. AD BDC-HFn	1419	88	952	58	2517
AD BDC-HFn vs. CTR BDC-HFn	29	7	52	10	98
AD Hfn-BDC vs. CTR NT	151	30	566	24	771
CTR NT vs. CTR BDC-HFn	164	26	724	22	936

**Table 2:** Most deregulated genes identified between AD-NT and CTR-NT and their suggested roles in AD.

	Most Deregulated Genes in AD-NT Group Compared to CTR-NT	
Gene	Fold Change	Role in AD
CXCL5	−5.28042797835226	monocytes migrating from blood to brain in AD patients [48]
CD1E	−3.76621256916995	CD1A is involved in longitudinal changes AD phenotypes [49]
IL12RB2	−2.36344776700486	associated with cognitive aging [50]
IL18RAP	−1.994385464	associated to Tau concentration in CSF of AD patients [51]
LILRA6	1.930851051	expressed in monocyte, function unclear [52]
LILRB5	4.048756658	expressed in monocyte, function unclear [52]
HLA-DRB6	3.84178295	associated to late onset AD [53]
TLR5	3.589139449	may regulate Aβ clearance [54]

**Table 3:** Baseline characteristics of the subjects recruited for this study.

	CTRs	AD
Recruited subjects	15	15
Age (mean ± SD)	56.1 ± 5.2	74.4 ± 8.8
Males %	43%	47%
Females %	57%	53%
MMSE	29.625 ± 0.74	18.5 ± 3.88

CTRs = controls; AD = Alzheimer’s Disease; SD = standard deviation.

## Data Availability

Not applicable.

## Supplementary Materials

The supporting information can be downloaded at: https://www.mdpi.com/article/10.3390/ijms23169237/s1.
