# Supplementary material for: Bisdemethoxycurcumin (BDC)-Loaded H-Ferritin-Nanocages Mediate the Regulation of Inflammation in Alzheimer’s Disease Patients
Source: Int J Mol Sci. 2022 Aug 17;23(16):9237. doi: 10.3390/ijms23169237 (PMC9409287; doi:10.3390/ijms23169237)
Supplement: Supplementary file 1 [file ijms-23-09237-s001.zip › BDC Ferritin Suppl Info v2.pdf]

Supporting Information

Bisdemethoxycurcumin (BDC)-Loaded H-Ferritin-Nanocages Mediate the Regulation of Inflammation in Alzheimer's Disease Patients

Stella Gagliardi [1], Marta Truffi [2], Veronica Tinelli [3], Maria Garofalo [1], Cecilia Pandini [1], Matteo Cotta Ramusino [1], Giulia Perini [1], Alfredo Costa [1,4], Sara Negri [2], Serena Mazzucchelli [5], Arianna Bonizzi [5], Leopoldo Sitia [5], Maria Busacca [1], Marta Sevieri [5], Michela Mocchi [2], Alessandra Ricciardi [2], Davide Prosperi [3], Fabio Corsi [2,3,4,5], Cristina Cereda [1] and Carlo Morasso [2,*]

[1] IRCCS Mondino Foundation, 27100 Pavia, Italy
[2] Istituti Clinici Scientifici Maugeri IRCCS Spa SB, 27100 Pavia, Italy
[3] Department of Biotechnology and Bioscience, University of Milano-Bicocca, 20126 Milano, Italy
[4] Department of Brain and Behavioral Sciences, University of Pavia, 27100 Pavia, Italy
[5] Department of Biomedical and Clinical Sciences, Università degli Studi di Milano, Via G. B. Grassi 74, 20157 Milano, Italy
* Correspondence: carlo.morasso@icsmaugeri.it

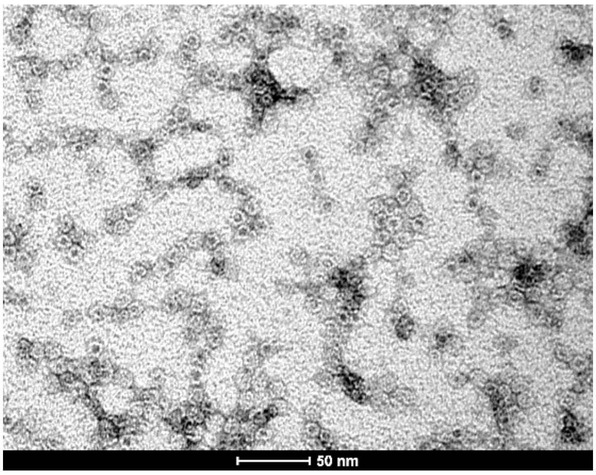

Figure S1. TEM pictures of HFn nanoparticles.

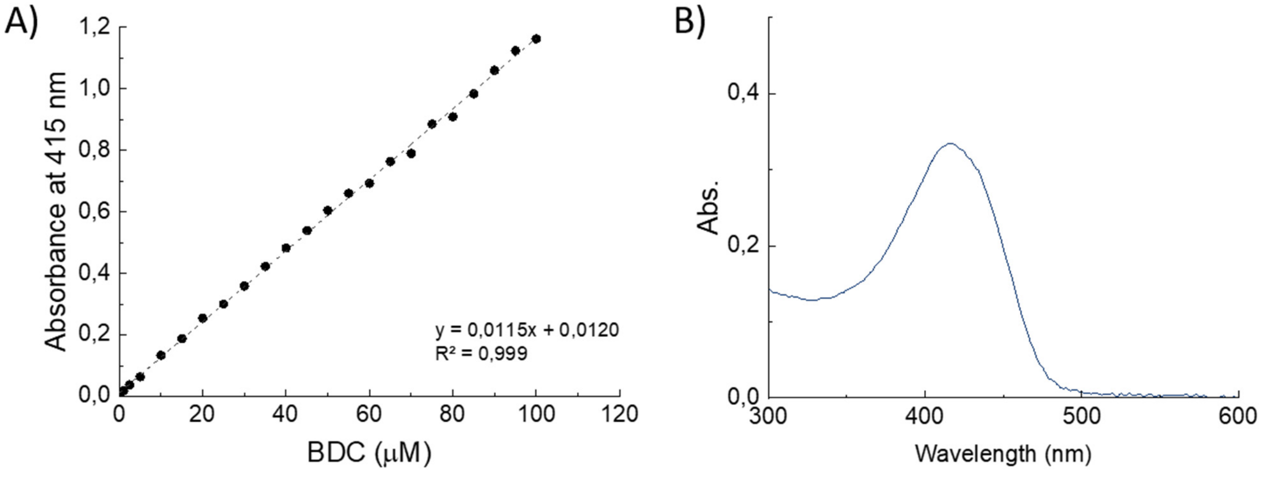

Figure S2. (**A**) Calibration line was obtained by measuring the spectra of standard solutions of BDC (from 0 to 100 μM) dissolved in acetic acid immediately after preparation. (**B**) UV-Vis spectrum of newly prepared solution of BDC-HFn.

A

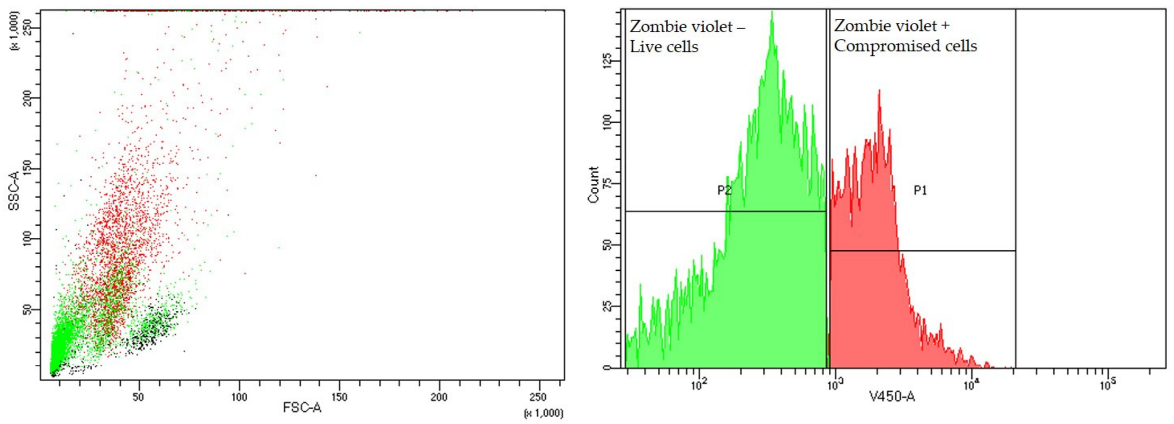

Population	#Events	%Parent	%Total
All Events	10,000	####	100.0
P1	3,157	31.6	31.6
P2	5,830	58.3	58.3

B

Total (cell/ml)	Live cells (cell/ml)	Cells viability (%)
3277777.78 ± 1651088.87	2865666.67 ± 1582375.27	83.89 ± 9.97

Figure S3. (**A**) PBMCs treated with Zombie Violet™ and analyzed through FACS, an amine-reactive fluorescent dye that is non-permeant to live cells but permeant to the cells with compromised membranes. (**B**) Table reporting cells counts through Trypan blue assay.

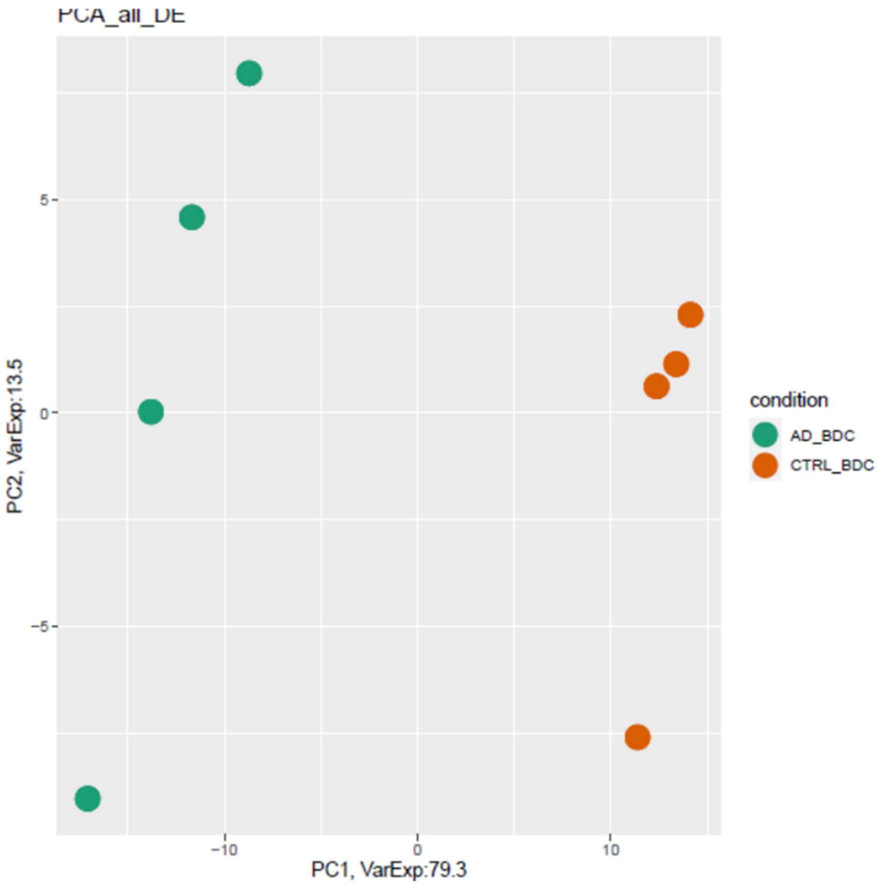

Figure S4. PCA of differentially expressed genes. All comparisons are given between AD BDC-HFn and CTR BDC-HFn.

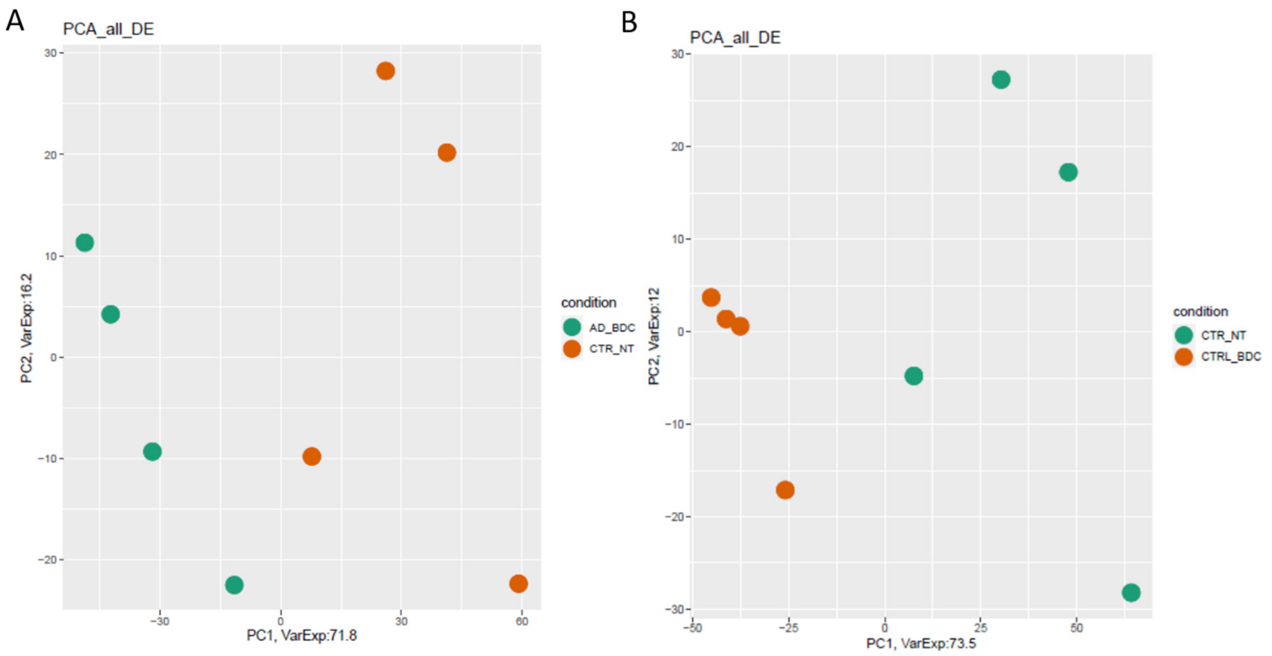

Figure S5. (**A**) PCA of differentially expressed genes. All comparisons are given between AD BDC-HFn vs. CTR NT. (**B**) CTR NT vs. CTR BDC-HFn.

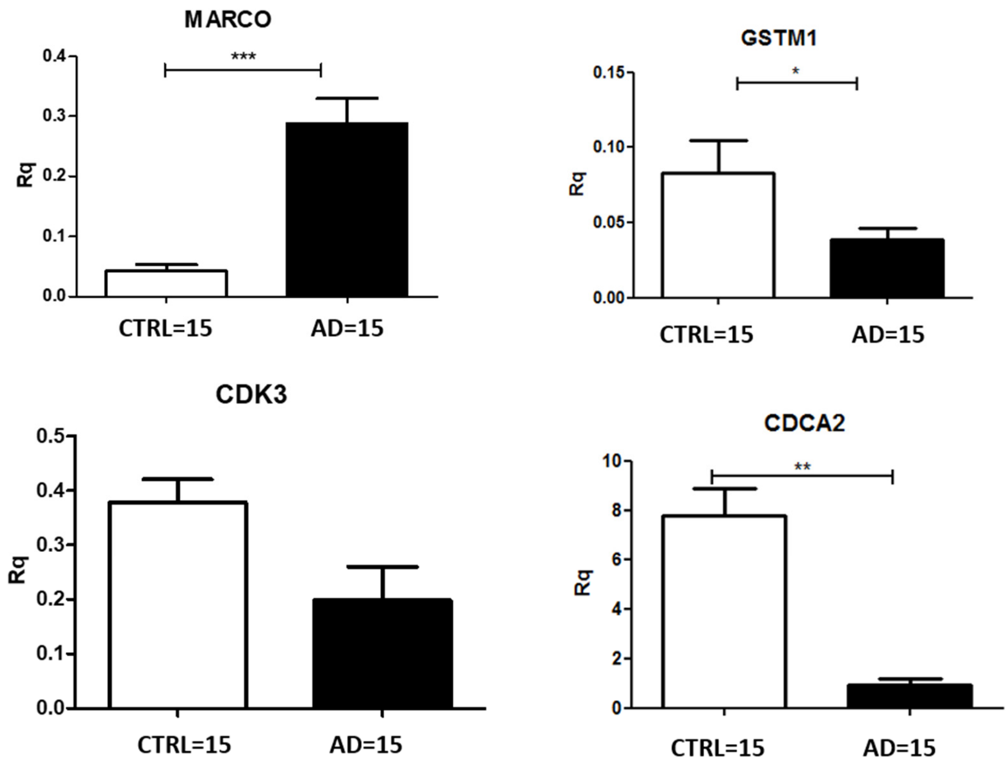

Figure S6. Differentially expressed transcripts verified by Real Time PCR in PBMCs from same RNA-seq cohort of AD and CTRs.

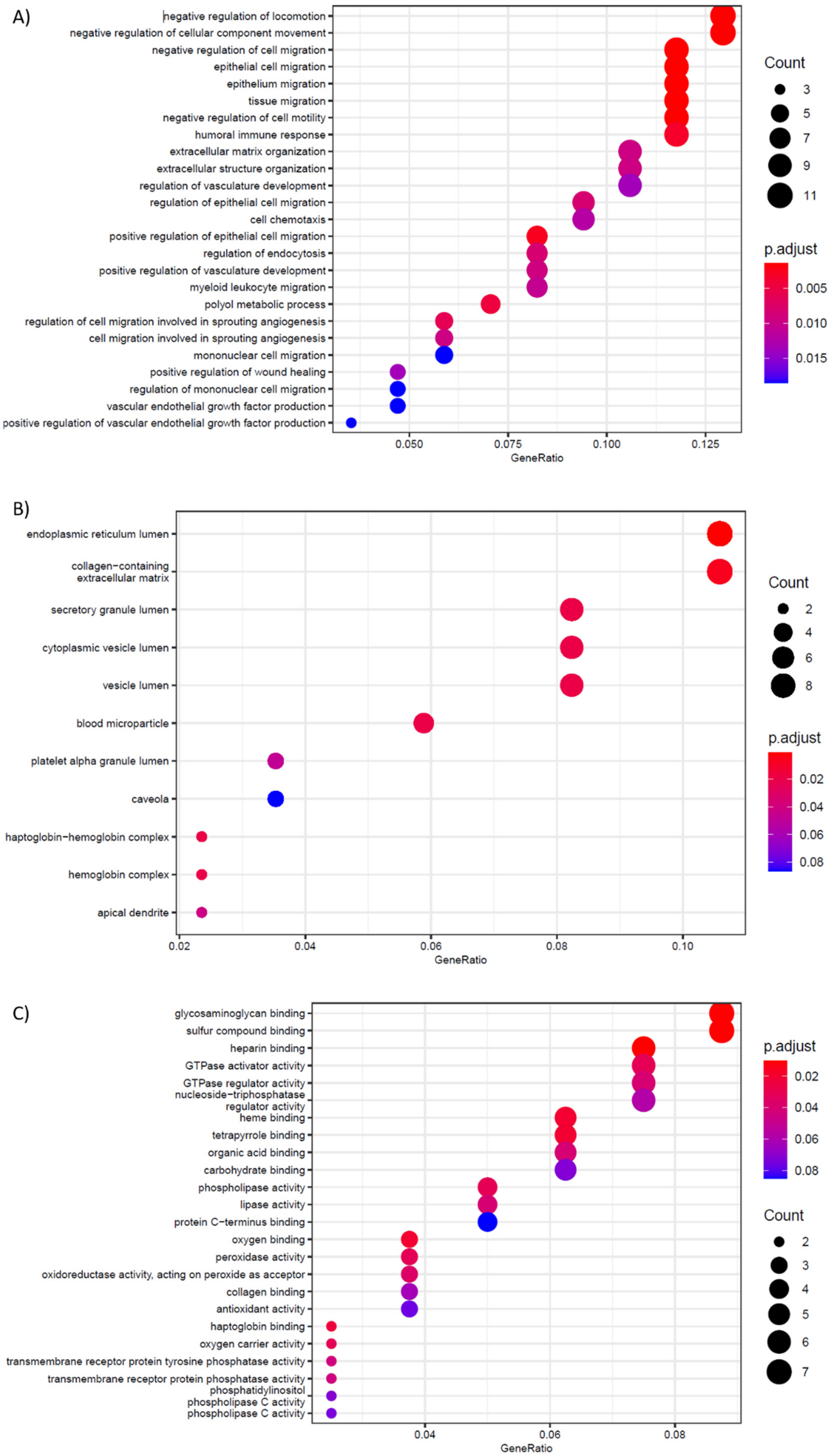
A)
negative regulation of locomotion
negative regulation of cellular component movement
negative regulation of cell migration
epithelial cell migration
epithelium migration
tissue migration
negative regulation of cell motility
humoral immune response
extracellular matrix organization
extracellular structure organization
regulation of vasculature development
regulation of epithelial cell migration
cell chemotaxis
positive regulation of epithelial cell migration
regulation of endocytosis
positive regulation of vasculature development
myeloid leukocyte migration
polyol metabolic process
regulation of cell migration involved in sprouting angiogenesis
cell migration involved in sprouting angiogenesis
mononuclear cell migration
positive regulation of wound healing
regulation of mononuclear cell migration
vascular endothelial growth factor production
positive regulation of vascular endothelial growth factor production
0.050
0.075
0.100
0.125
GeneRatio
Count
3
5
7
9
11
p.adjust
0.005
0.010
0.015
B)
endoplasmic reticulum lumen
collagen−containing extracellular matrix
secretory granule lumen
cytoplasmic vesicle lumen
vesicle lumen
blood microparticle
platelet alpha granule lumen
caveola
haptoglobin−hemoglobin complex
hemoglobin complex
apical dendrite
0.02
0.04
0.06
0.08
0.10
GeneRatio
Count
2
4
6
8
p.adjust
0.02
0.04
0.06
0.08
C)
glycosaminoglycan binding
sulfur compound binding
heparin binding
GTPase activator activity
GTPase regulator activity
nucleoside−triphosphatase regulator activity
heme binding
tetrapyrrole binding
organic acid binding
carbohydrate binding
phospholipase activity
lipase activity
protein C−terminus binding
oxygen binding
peroxidase activity
oxidoreductase activity, acting on peroxide as acceptor
collagen binding
antioxidant activity
haptoglobin binding
oxygen carrier activity
transmembrane receptor protein tyrosine phosphatase activity
transmembrane receptor protein phosphatase activity
phosphatidylinositol phospholipase C activity
phospholipase C activity
0.04
0.06
0.08
GeneRatio
p.adjust
0.02
0.04
0.06
0.08
Count
2
3
4
5
6
7

Figure S7. AD BDC-HFn versus CTR BDC-HFn. GO enriched terms for Biological process (**A**), cellular component (**B**) and molecular function (**C**).

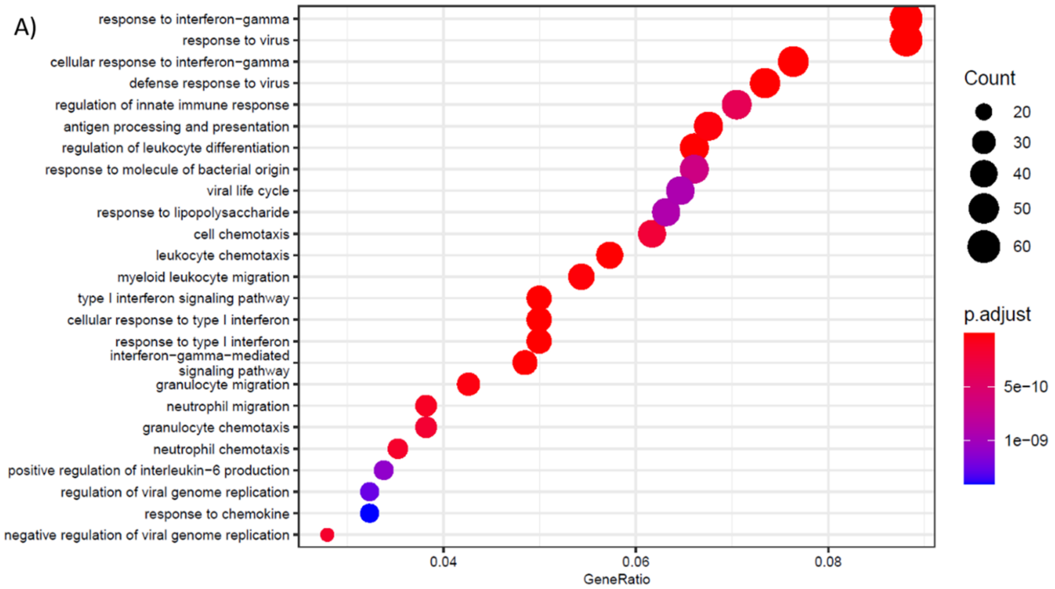

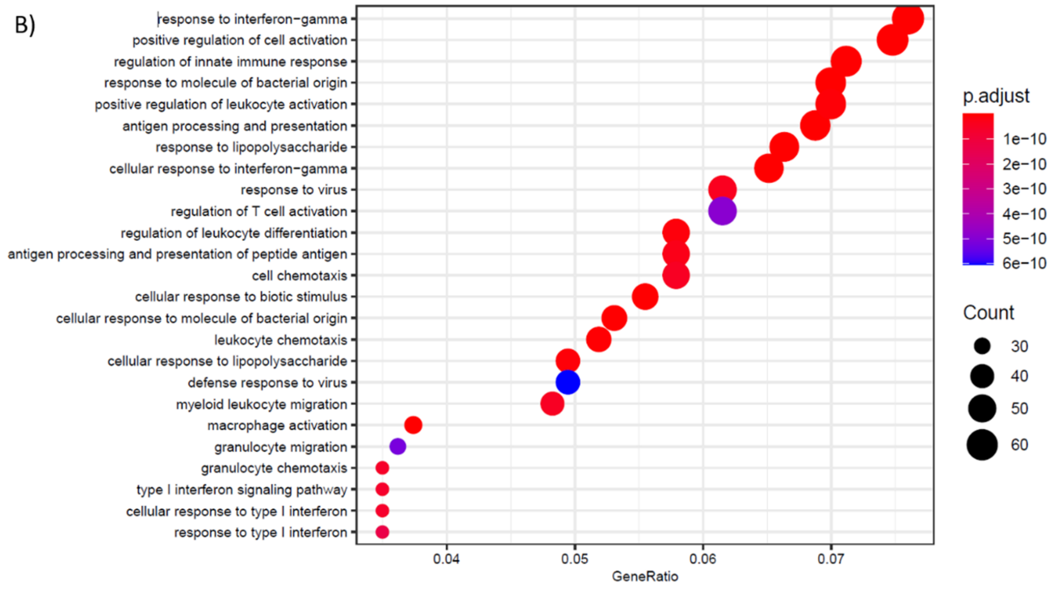

Figure S8. (**A**) AD BDC-HFn vs CTR NT Biological process. (**B**) CTR NT vs CTR BDC-HFN Biological process.

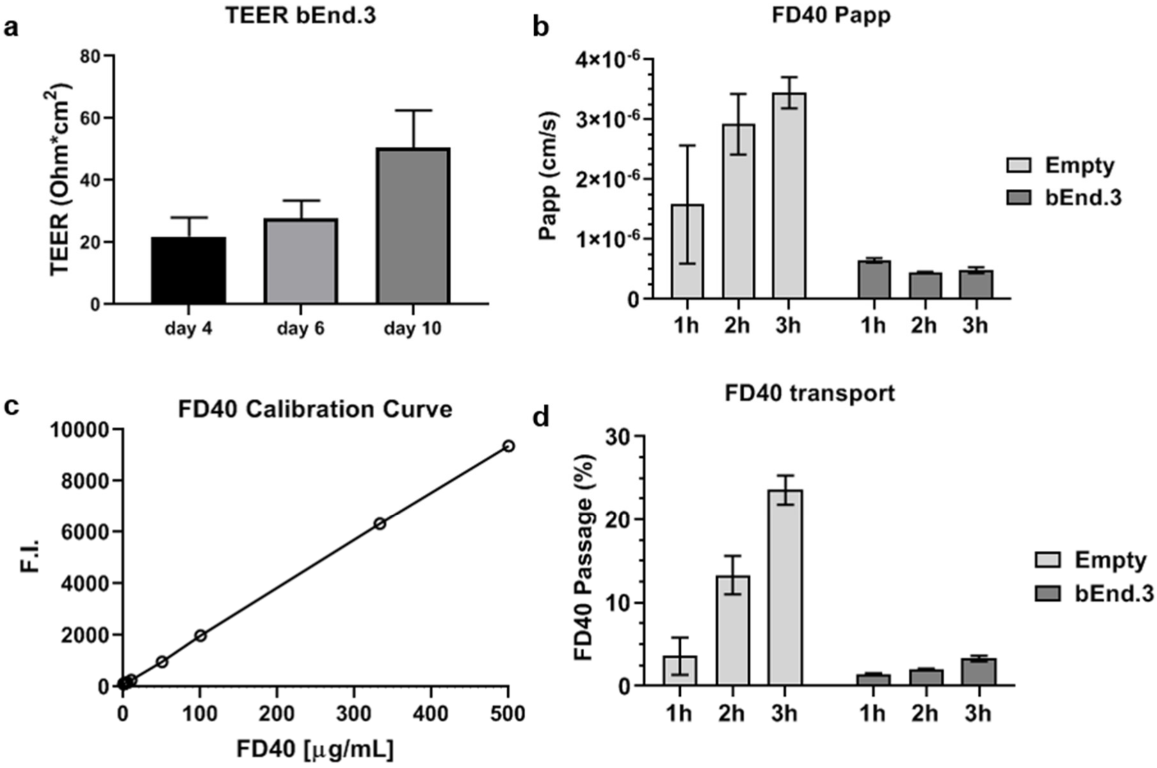

Figure S9. Characterization of bEnd.3 monolayer: TEER values measured 4, 6 and 10 days after seeding cells on transwell membranes (**a**). Analysis of FD40 permeability evaluated as Papp (**b**) based on a calibration curve obtained with FD40 at different concentrations (**c**); ratio between the measured concentration of FD40 on the basolateral side and the concentration at equilibrium (**d**). Measurements were run at day 10, when TEER reached its maximum.

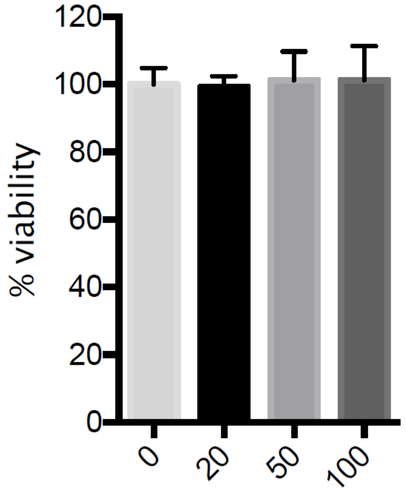

Figure S10. Viability of bEnd.3 cells upon treatment with 20, 50, 100 µg mL^{-1} of BDC-HFn for 24 h. Viability data are reported as mean percentage ± standard deviation of 6 replicates after normalization on untreated cells (light-gray histogram on the left, 100% viability).
